# Fuzzy-based hunger games search algorithm for global optimization and feature selection using medical data

**DOI:** 10.1007/s00521-022-07916-9

**Published:** 2022-11-01

**Authors:** Essam H. Houssein, Mosa E. Hosney, Waleed M. Mohamed, Abdelmgeid A. Ali, Eman M. G. Younis

**Affiliations:** 1 Faculty of Computers and Information, Minia University, Minia, Egypt; 2 Faculty of Computers and Information, Luxor University, Luxor, Egypt

**Keywords:** Hunger games search (HGS), Feature selection (FS), Metaheuristic algorithms (MAs), Quantitative structure-activity relationship (QSAR).

## Abstract

Feature selection (FS) is one of the basic data preprocessing steps in data mining and machine learning. It is used to reduce feature size and increase model generalization. In addition to minimizing feature dimensionality, it also enhances classification accuracy and reduces model complexity, which are essential in several applications. Traditional methods for feature selection often fail in the optimal global solution due to the large search space. Many hybrid techniques have been proposed depending on merging several search strategies which have been used individually as a solution to the FS problem. This study proposes a modified hunger games search algorithm (mHGS), for solving optimization and FS problems. The main advantages of the proposed mHGS are to resolve the following drawbacks that have been raised in the original HGS; (1) avoiding the local search, (2) solving the problem of premature convergence, and (3) balancing between the exploitation and exploration phases. The mHGS has been evaluated by using the IEEE Congress on Evolutionary Computation 2020 (CEC’20) for optimization test and ten medical and chemical datasets. The data have dimensions up to 20000 features or more. The results of the proposed algorithm have been compared to a variety of well-known optimization methods, including improved multi-operator differential evolution algorithm (IMODE), gravitational search algorithm, grey wolf optimization, Harris Hawks optimization, whale optimization algorithm, slime mould algorithm and hunger search games search. The experimental results suggest that the proposed mHGS can generate effective search results without increasing the computational cost and improving the convergence speed. It has also improved the SVM classification performance.

## Introduction

Optimization is a procedure for maximizing or minimizing an objective function or multiple objectives [1]. Many problems can be handled by employing several optimization techniques to find the optimum solution. Optimization is used to create optimal paths in real life. Metaheuristics are useful tools that provide different ways of creating effective optimization algorithms. Although the exact solution is not offered, the algorithm can provide the best possible solutions. Feature selection (FS) is one of the most important preprocessing steps in data mining and pattern recognition. Its aim is to filter features and select a subset from a given training dataset. One of the benefits of FS is that it reduces the training time for model creation, eliminates overfitting, and improves the generalization of the models for a variety of datasets, such as biomedical signal processing, medical images, DNA microarray data, chemical data, and drug development. The feature dimensions of the data acquired from multiple medical sources are incredibly high. Relevant literature has demonstrated that applying FS to various medical domain data has a considerable effect on the results. There are various methods for FS used in machine learning (ML) and data mining [2]. They can be divided into filtering, wrapper, and embedding (hybrid method).

First, filtering techniques evaluate each feature subset using an objective function based on target relevance or feature correlation. Second, wrapper approaches explore a feature subset based on a predefined classifier performance score. Third, hybrid method is the combination of filter and wrapper method that is built by the algorithm has built-in FS methods. Example of this method ridge regression has penalization inbuilt. FS can be considered an NP hard problem as there are many possible solutions, especially for large feature space [3]. A binary version of several metaheuristic algorithms (MAs), such as wrapper techniques for FS, has been proposed to provide a suitable solution. Some MAs such as genetic algorithm (GA) [4], particle swarm optimization (PSO) [5], bee colony optimization (BCO) [6], cuckoo search (CS) [7], grey wolf optimizer (GWO) [8], improved multi-operator differential evolution algorithm (IMODE) [9], gravitational search algorithm (GSA) [10], Harris’ Hawks optimization (HHO) [1], whale optimization algorithm (WOA) [11], and slime mould algorithm (SMA) [12] have been applied for FS and support vector machine (SVM) kernel parameters are optimized simultaneously [13]. FS has been widely used in sequence analysis for Bioinformatics. Content and signal analysis is two sorts of challenges that can be solved using FS. The content analysis examines a sequence’s general properties, such as its propensity to code for proteins or its ability to perform a certain biological function. In contrast, the signal analysis identifies key motifs in the sequence as regulatory elements or gene structural elements.

This study proposes a modified hunger games search (mHGS) hybrid metaheuristic algorithm to solve the problems of classical HGS optimization algorithm. It simulates the hungry behaviour in animals. For FS, mHGS algorithm performance was assessed by well-known benchmark test functions and a set of well-known medical datasets. The fitness function is based on a straight-forward understanding of hunger as a critical biological drive. The fitness value for hunger is higher than other compared algorithms. Other hunger games can alter their initial positions based on the fittest hunger game. These behaviours can be related to the goal function to be optimized. The mHGS effectiveness has been tested on the complex CEC’20 benchmark functions and several biomedical datasets, and compared with several counterpart MHAs including IMODE, GSA, GWO, HHO, WOA, SMA, and original HGS algorithm [14]. The experimental results proved that the proposed mHGS has a stronger search capability than the basic HGS, and some state-of-the-art MH methods.

*Motivation* Despite eminent applications, the hunger games search (HGS) has attracted the attention of many researchers—the method has reached more than 200 citations in about a year. HGS is still attributed for its slow convergence and stagnancy issues when employed on high-dimensional problems [15]. It sometimes generates low-diversified solutions towards the end of iterations, which causes a situation for the search agents trapped in local optima. Additionally, its exploration phase is highly dominant with extraordinary randomization that seems clueless search mechanism. The promising outcomes of several HGS-based hybrid approaches proposed in the literature, especially HGS, have been carried out with chaotic maps in three alternative scenarios [16]. Additionally, HGS is merged with the food-searching techniques of the whale optimization algorithm (WOA) for global optimization [17]. In the same context, in order to identify unknown parameters of solar photovoltaic (PV) systems, the Nelder-Mead simplex method (NMs) is injected in HGS to increase the population diversity [18].

In this study, the proposed mHGS algorithm integrates the efficacy of fuzzy into HGS’s exploration phase. Moreover, additional modifications are also proposed to select animals. The enhanced HGS variant is not only tested on some classification datasets (with feature-size greater than 15000) for feature selection problem, also it is evaluated on hard numerical optimization problems presented in CEC’20 test suite which is well-known in optimization community for its difficult search space. The outcome of the simulations performed in this research reveals superiority of the proposed approach when compared with the conventional HGS and other optimization algorithms, as well as, several other counterparts introduced recently; however, it lags superiority on state-of-the-art methods like improved multi-operator differential evolution algorithm (IMODE) [9], gravitational search algorithm (GSA) [10], grey wolf optimization (GWO) [8], Harris Hawks optimization (HHO) [1], whale optimization algorithm (WOA) [11], and slime mould algorithm (SMA) [12]. The proposed mHGS maintains trade-off balance between exploration and exploitation, convergence speed, and better global search ability. The argument is well supported with various statistical measurements and evaluation metrics later in this paper.

In summary, the main contributions of this paper are as follows:

- The traditional HGS is enhanced by adding the following mechanism: Fuzzy logic-based mutation for control parameters strategy, balancing exploration/exploitation strategy, and population reduction strategy.
- mHGS is proposed as an alternate feature selection approach.
- mHGS is proposed to improve its local search capability and solve the problem of premature convergence.
- The proposed mHGS algorithm achieved superior results compared to its counterparts.
- Various metrics from statistical to qualitative analyses assess the performance of the proposed mHGS algorithm.

The following is how the rest of the paper is organized: Sect. 2 presents the related work, highlighting several recent related research. Preliminaries on QSAR approach, hunger games search (HGS), and support vector machine (SVM) are introduced in Sect. 3. Section 4 discusses the proposed mHGS algorithm including the fuzzy logic basics, fuzzy logic-based mutation, membership function formation (MFs), centroid-based fuzzy mutation, fitness function (fobj), pseudo-code of mHGS algorithm, and mHGS development phases. Section 5 introduces two experimental series, including the CEC’20 benchmark functions, feature selection (FS) and discussion. Finally, Sect. 6 shows the conclusions and future work.

## Related research

Here, we highlight some of the important research in line with the proposed work. In [19], for creating a medical molecule, diagnosing several diseases, or determining the optimal drug, so it is necessary to collect relevant data. There are many ways of collecting medical or chemical data. First, for medical molecules, protein bank and ZINC databases are used to select the suitable crystal of protein structure. One of the most efficient methods for making drugs from chemical data is computer-aided drug design (CADD). In [20], CADD is used to identify drug design which can be classified into quantitative structure-activity relationship (QSAR) and docking. The binding between protein and ligand is docking. Many chemical compounds can be extracted from the Pubchem website.

In [21], proteins and ligands are tied with each other, so ligands must be separated from the protein. The best drug depends on the best ligands with less energy. Pymol software is used for the separation operation. AutoDocks software is used to calculate the energy for several ligands [22].

In [23], multiple tasks have been proposed with a drug review dataset. Sentiment analysis is used to predict user sentiments about medicine side effects and effectiveness based on user reviews. The transferability of trained classification models among domains has been investigated to overcome several challenges for lacking annotated data. The transfer learning approaches have been proposed to indicate the similarities across different domains. For all prediction tasks, classification-based sentiment analyses, as an n-grams approach, are applied to indicate several user reviews. The classification model for this dataset achieves 92.24% accuracy. A method based on biclustering has been discussed in [24] to reduce the molecular descriptors number to predict chemical compounds biodegradation. Several classifiers were used to assess the biodegradation task. For the QSAR biodegradation data, the testing results indicate that random forest is the best classifier, with an accuracy of 88.81% and only 19 MD.

In [25, 26], artificial intelligence (AI) has made significant progress, allowing the development of various horizons for QSAR modelling based on machine learning. In [27], some authors proposed combining artificial neural networks (ANN) and support vector machines (SVM) for QSAR modelling, with PCA used to minimize data dimensionality. The performance is measured on the QSAR Biodegradation dataset, with an accuracy 82%. In [28], silico models have been described to identify the organic AR modulators. ML methods predicted AR binding based on the tree classification model as k-nearest neighbour (k-NN), RF, and naive Bayes. The models achieved robust and reliable predictions.

In [29], the optimum treatment for the two most frequent forms of warts, plantar and common has been determined in response to two of the best treatment modalities such as immunotherapy and cryotherapy). The treatment approach was chosen at random using fuzzy rule-based inference mechanism to forecast treatment technique response. The percentages for immunotherapy and cryotherapy were 83.33% and 80.7%, respectively. Moreover, such expert systems reduce treatment costs, save time for patients, and also improve treatment quality. In [30], two peptide datasets have been used; one for lung cancer cells and the other for breast cancer cells. ANN has also been used for recognizing the peptides inducing breast/lung cancer death. Fourteen peptides from 1000 denovo designs were selected for in vitro testing and production on breast cancer. In [31], it has been indicated as the most frequent cause of dementia in the world, Alzheimer’s disease. Due to the timeliness of diagnosis and the ageing of the population, it has begun to outrun. Due to a lack of sensitivity and precision, case classification, magnetic resonance imaging, and neuropsychological testing are ineffective. The convolutional neural network has been used to construct a framework to indicate many Alzheimer’s disease features.

In [32], FS is divided into four steps: (1) select the appropriate features, (2) analyse the subset using various metrics, (3) locate other sets, and (4) feature validation. In [33, 34], it has been shown that wrapper-based solutions outperform filter approaches in terms of results. The wrapper approach is more time-consuming, yet yields precise results. FS may be tweaked to be more efficient by identifying the best subset of attributes to tackle a range of problems. MHAs have many advantages.

The authors in [35] suggest that the wrapper-based techniques have attracted significant attention due to the use of learning algorithms which influence the selection of significant features. In [36], the MHA-based approach SSA using wrapper FS has been presented to estimate chemical compound activity by determining the most suitable subset of molecular descriptors from the MAO dataset. SSA is compared with many MHAs, including the moth-flame optimization algorithm (MFO), grasshopper optimization algorithm, and sine cosine algorithm. It is worth noting that SSA with the k-NN classifier had the best accuracy of 87.35% while keeping 783 chemical descriptors. In [37], two classification approaches, HHO-SVM and HHO-kNN, for drug design and discovery prediction have been proposed. Several techniques for FS are explained. In [38], the strategies for selecting medications based on their features and the importance of chemical descriptors have been presented. FS can be considered a multi-objective optimization problem by decreasing the number of the selected features and increasing the accuracy.

In [39], some authors described the FS approach used in medication development. During the FS phase, crude set-based approaches are used to identify the most discriminative features. The most discriminative traits were chosen using rough set-based approaches. Several features were selected from the feature vector at this step using three distinct rough set-based methods, such as QRFS, DMFS, and EBFS. Using these algorithms has the goal of reducing the number of features to improve classification performance and reduce classification time. In [40], the authors suggested FS techniques used in various domains, primarily to deal with data with high dimensions. Several FS solutions based on (MHAs) methods have been developed to address the FS problem and overcome the limits of classic FS approaches. Also, in [41], the authors outlined how ML system requires FS. The effectiveness of such systems heavily depends on the relevancy of features to the target. FS is an NP-hard problem because there are many alternative solutions, especially for large feature space. A novel multi-population-based PSO (MPPSO) has been presented for FS.

In [42], MHAs are used to determine important features to boost the performance of high-dimensional datasets to devise efficient knowledge extraction systems declared FS as an important preprocessing step that helps to avoid the effect of noisy, and inconsistent features on model performance. However, when used with dataset with several features these algorithms frequently suffer from a local optimally problem because of the large solution space. In [43], a unique technique for dimensionality reduction has been used to improve classification accuracy, which uses the Henry gas solubility optimization process for picking relevant features. In [44], many feature extraction approaches have been presented to test their prediction performance. During the testing step, it will be necessary to specify the facial picture view and the recorded eye-gaze locations. Table 1 depicts some published literature studies on related topics.

**Table 1:** A review of previously proposed methods

References	Year	Algorithms	Datasets	Accuracy
[45]	2021	The Fuzzy Support Vector Machines were used to propose a PCA approach (FSVM)	Microarray cancer datasets	With 96.92% accuracy that only 60
[46]	2020	A Crow Search Optimization Algorithm with Chaos Theory and a Fuzzy C-Means Algorithm were implemented	Ten medical datasets	Medical datasets performance has improved
[47]	2014	Fuzzy logic and Fisher’s Linear Discriminant Analysis (FDA)	MIT-BIH database	Fuzzy logic and Fisher’s LDA approach accuracy, respectively, is 94.03% and 93.87%
[48]	2016	Using the hybridized filter-wrapper technique, a fuzzy with multi-objective fs (FC-MOFS) was developed	Six datasets	They provide higher accuracy and feasibility results
[49]	2019	The Immune Optimization Algorithm (IOA) is used in hybrid with the Fuzzy Support Vector Machine (FSVM) (FSVM-IOA)	Heart-Disease datasets	According to the FSVM-IOA accuracy, the forward FSVM-IOA accuracy is 95.82% and the reverse FSVM-IOA accuracy is 96.01%
[50]	2021	HHOFKNN uses Harris Hawks Optimization (HHO) to optimize the Fuzzy K-Nearest Neighbour (FKNN)	COVID-19 dataset	prediction accuracy and stability, the HHO-FKNN technique surpasses conventional machine learning algorithms
[51]	2021	Multi-Objective Artificial Bee Colony with Fuzzy Mutual (MOABC)	Six datasets	MOABC is a tool that can be used to tackle feature selection challenges

## Preliminaries

The basic QSAR methodologies and structure of hunger game optimization and SVM are explained in this section.

### Quantitative structure-activity relationship (QSAR)

QSAR is used to express the chemical structure and the biological activity relationship in a mathematical form. It is useful, especially when it is used to recognize the chemical compounds’ features. Many ML algorithms have been successfully used to analyse structure-activity relationship to predict whether a substance is similar to a drug-like activity. Many complicated molecular compounds can be applied to characterize variety of properties. In chemistry and pharmacology, molecular descriptors are crucial [52]. QSAR techniques are based on developing statistical models for the association between chemical structure and biological activity. The classification process has been used in the chemical-biological interaction between many biomolecules.

In QSAR studies, the next step is to develop a statistical approach using the descriptors obtained previously from several compounds. This model’s primary purpose is to predict activity for new compounds and to use it for understanding the action predicted for a specific drug. The input data accuracy, descriptors and statistical techniques selection, and produced model validation play a significant role in the quality of a QSAR model [53]. In the same context, the ANNs and SVMs are applied in the QSAR field and other molecular modelling approaches have recently attracted significant attention as key tools in drug discovery as discussed in [54, 55].

### Hunger games search (HGS)

The HGS algorithm is discussed in this section, with its mathematical model [14]. The HGS simulates the hunger behaviour of selected animals. HGS fitness is determined by the approach used to track hunger behaviour as a critical homeostatic incentive. Many behaviours that generate action and choice in the animals’ lives are used to confine and understand the optimization process for new uses. The algorithm’s feature process is an adaptive weight based on the hunger concept used and built to replicate each hunger effect search stage. Most animals’ computationally used logical principles and game activities are adaptive evolutionary chances for food acquisition. The basic notion is that the proposed technique is more efficient because of its high performance, dynamic nature, and simple structure in terms of convergence and quality for acceptable solutions. HGS performance was compared with other optimization algorithms with several experimental results such as CEC’20 benchmark.

#### Approach food

Mathematical formulas express the behaviour of approach food, and the following procedures are proposed to simulate the contraction mode. The mathematical formulas Eq. (1) is as follows:

1 $$ \overrightarrow {{{\text{fobj}}(D,G,X(t + 1))}}= \left\{ {\begin{array}{*{20}l}{\overrightarrow {{{\text{fobj}}(D,G,X(t))}}\cdot (1 + {\text{randn}}(1)),\;r_{1}< l} \hfill\\{\overrightarrow {{W_{1} }}\cdot \overrightarrow {{X_{b} }}+ {\mathbf{R}} \cdot \overrightarrow {{W_{r} }}\cdot \left| {\overrightarrow {{{\text{fobj}}(D,G,X_{b} )}}- \overrightarrow {{{\text{fobj}}(D,G,X(t))}} } \right|,\;r_{1}> l,r_{r}> E} \hfill\\{\overrightarrow {{W_{1} }}\cdot \overrightarrow {{{\text{fobj}}(D,G,X_{b} )}}- {\mathbf{R}} \cdot \overrightarrow {{W_{r} }}\cdot \left| {\overrightarrow {{{\text{fobj}}(D,G,X_{b} )}}- \overrightarrow {{{\text{fobj}}(D,G,X(t))}} } \right|,\;r_{1}> l,r_{r}< E} \hfill\\ \end{array} } \right. $$

where *R* is within $[-a,a]$; $r_1$ and $r_2$ represent random numbers within [0,1]; $W_{1}$ and $W_{2}$ denote hunger weights; $X_b$ denotes a random person in the population; and *X*(*t*) is an individual. The formula of Eq. (2) is as follows:

2 $$\begin{aligned} E={\text {sech}}(|F(i)-{\hbox {BF}}|) \end{aligned}$$

where $i \in 1, r, \ldots , n, F(i)$ is the fitness for an individual *i*, whereas the best fitness value for the current iteration is denoted by BF. The hyperbolic function (Sech) is represented as (3):

3 $$\begin{aligned}&\left( {\text {sech}}(x)=\frac{r}{e^{x}+e^{-x}}\right) \end{aligned}$$

4 $$\begin{aligned}&\mathbf {R}=r \times a \times \text{ rand } -a \end{aligned}$$

#### Hunger role

Equations (5) and (6) represent the mathematical formulation of the role:

5 $$ \overrightarrow {{W_{1} (i)}}= \left\{ {\begin{array}{*{20}l}{{\text{hungry}}(i) \cdot \frac{N}{{{\text{ SHungry }}}} \times r_{4} ,r_{3}< l} \hfill\\{1r_{3}> l} \hfill\\ \end{array} } \right. $$

6 $$\begin{aligned}&\overrightarrow{W_{2}(i)}=(1-\exp (-\mid {\text {hungry}}(i)- \text{ SHungry } \mid )) \times r_{5} \times 2 \end{aligned}$$

where the population of individuals is denoted by *N*, whereas SHungry is sum(hungry). The randomization in search is injected using random variables $r_{3}$, $r_{4}$ and $r_{5}$. Equation (7) is a formulation for hungry(i):

7 $$\begin{aligned} {\text {hungry}}(i)=\left\{ \begin{array}{lr} \dot{ }&{} \text{ AllFitness } (i)=={\hbox {BF}} \\ _{\text{ hungry } }(i)+H, &{} \text{ AllFitness } (i) !={\hbox {BF}} \end{array}\right. \end{aligned}$$

where AllFitness(i) preserves each individual’s fitness in the current iteration.

8 $$\begin{aligned}&{\hbox {TH}}=\frac{F(i)-{\hbox {BF}}}{{\hbox {WF}}-{\hbox {BF}}} \times r_{6} \times 2 \times ({\hbox {UB}}-{\hbox {LB}}) \end{aligned}$$

9 $$\begin{aligned}&H=\left\{ \begin{array}{cc} {\hbox {LH}} \times (1+r), &{} {\hbox {TH}}<{\hbox {LH}} \\ {\hbox {TH}}, &{} {\hbox {TH}} \ge {\hbox {LH}} \end{array}\right. \end{aligned}$$

where $r_{6}$ is another randomization variable and *F*(*i*) indicates each individual’s fitness value. The best fitness and worst fitness values are denoted by BF and WF, whereas the upper and lower search bounds are UB and LB, respectively. Because the hunger sensation *H* has a lower bound, LH, it contributes to the algorithm’s optimum performance.

### Support vector machine

It is a supervised learning techniques applied in classification tasks [56]. Using the core concepts for the nonlinear kernel function, the approach is used to map data from a high-dimensional space. To identify the optimum solution for separating two classes, the SVM is utilized. Regression and classification problems are solved by the SVM model. The basic principle behind SVM is that the algorithm generates a hyperplane that is used to divide data into classes. SVMs’ first major challenge is to find a dividing line (or hyperplane) between data from two classes. SVM is a classification technique that uses data to produce a line that divides the classes. It aims to optimize different margins in the closest positions, referred to as support vectors and hyperplanes. The algorithm output is an optimal hyperplane. This hyperplane is a two-dimensional line that splits a plane into two sections, each with its own class.

In the SVM result, various parameters are controlled. The arguments determined by the designer when the classifier is formed are referred to as tuning parameters. The *C* parameter regulates the balance between a smooth decision boundary and correctly identifying training points. If *C* a large value is defined, it will appropriately obtain more training points, but it will also result in more convoluted decision curves attempting to fit all of the points into the output. To create a properly balanced curve and avoid overfitting, multiple values of C were used for the same dataset. Meanwhile, $\varGamma$ describes the range of influence of a single training session. If it has a low value, every point will have a long reach, and if it has a high value, every point will have a close reach. If $\varGamma$ has a very high value, then the decision boundary will be based solely on the points that are extremely close to the line, essentially ignoring some of the points that are quite far from the decision boundary. This is due to the fact that the points that are closer have a greater weight. If the $\varGamma$ value is low, even the far points gain a high weight.

SVM is a supervised learning technique used in classification [56]. Using the core concepts for the nonlinear kernel function, the svm approach is used to map data from a high-dimensional space. The SVM is utilized to identify the optimum solution for separating two classes. The SVM represents a linear model for regression and classification problems. It is an efficient method for various real situations and can solve both linear and non-linear problems. The basic principle behind SVM is that the algorithm generates a line or hyperplane that is used to divide data into classes. SVMs’ first major challenge is to find a dividing line (or hyperplane) between data from different classes. It aims to optimize different margins in the closest positions, referred to as support vectors and hyperplanes. The algorithm output is an optimal hyperplane. This hyperplane is a two-dimensional plane line that divides a plane into two sections, each class on either side. In the SVM result, various parameters are controlled. The arguments determined by the designer when the classifier is formed are referred to as tuning parameters.

In cheminformatics, SVM is one of the most widely used machine learning algorithms. The prediction of toxicity-related qualities such as mutagenic toxicity and toxicity categorization is one of the uses of SVM.

**Fig. 1:**
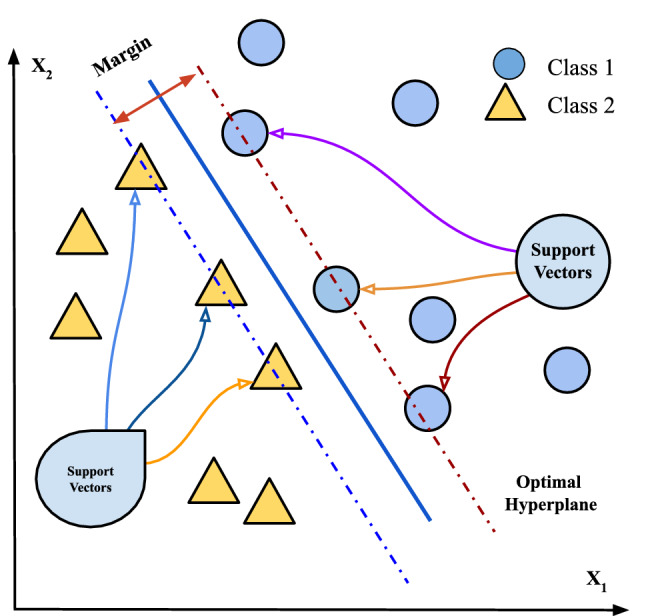
SVM classification algorithm implementation

SVM algorithm is introduced in Algorithm 1, and also Fig. 1 is used for declaration.

A graphical explanation is shown in Fig. 1.

**Figure:**
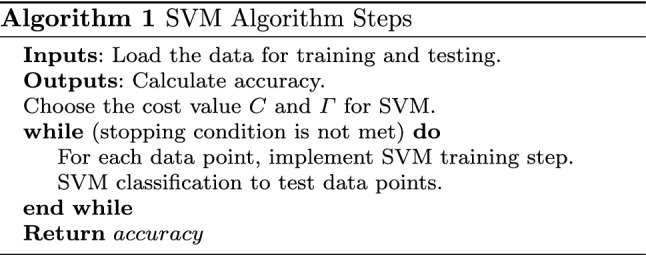

## The proposed mHGS

The proposed mHGS algorithm has been developed to address the HGS problem, which includes being caught in sub-optimal regions, delayed convergence, and is commended balance between global and local search, illustrated in convergence curves in Fig.4. Our enhancement strategy is based on two different testing methods,including CEC’20 and biological and chemical data with various evaluation criteria.

### Fuzzy logic

The fuzzy set theory [57] was introduced in 1965. It is used increasingly and employed in several domains, including image segmentation [58], cancer classification [59], and optimization [60]. Most natural objects cannot be defined because of simple shapes. Fuzzy logic’s characteristics are based on the truth value of a variable as a real number between 0 and 1. Set theory’s fuzziness is depicted graphically for future reference. Membership functions (MFs) can be used to define it. In the universal domain, any fuzzy set Eq. (10) is a set of ordered pairs as follows:

10 $$\begin{aligned} F=\left\{ \left( x, \mu _{F}(x)\right) \mid x \in U\right\} \end{aligned}$$

where x is a universal set of *U* element knowledge ; $\mu _{F}$ is the MF for *F* with values in [0, 1]. In the current domain, the existence of MF is an expertise element that can have many membership levels. Although the fuzzy set boundary is a nonzero element, it is an incomplete membership $(<1)$.

### Fuzzy logic-based mutation

The fuzzy logic notion is applied to address various research challenges in industrial applications. An MF indicates the membership value for an operation. Any fuzzy set can be defined as a collection of ordered pairs in the universal domain. Here, the MF of its $\hbox {i}{\hat{\hbox {t}}}\hbox {h}$ value is in the range [0, 1], and it is a universal set information element. Consequently, one element of information can have multiple degrees of membership in the current domain, depending on the nature of the MF. The fuzzy set’ core comprises of elements with full membership, where as support comprises of elements with nonzero membership. The fuzzy set’s boundary comprises of elements with nonzero but in complete membership.

### Membership functions formation (MF)

In fuzzy logic, MF plays a crucial role in the performance of fuzzy representations in different situations [57]. To be specific, the MF shape is critical for a certain problem because it controls the fuzzy inference rules. MF can be Gaussian, triangular, trapezoidal, or take other forms with their requirement that an MF’s values are between 0 and 1. MF basically maps the given data to the necessary degree of membership. A thorough study of the underlying problem can lead to the conclusion that the MF shape is appropriate for the application under certain conditions. There could be an infinite number of ways to define fuzziness. The approach depends on the nature of the problem. In addition to determining the MF shape, determining the interval and number of MFs is critical. Therefore, to regulate the system model in temperature terms using fuzzy logic, it is necessary to identify numerous MF (high, medium, and low) membership value intervals. These variables have a substantial impact on the inference of a fuzzy logic-based system. Observing data distribution is also a significant component. The trial and error method is sometimes employed to choose a MF form. The function can use any curve, as long as it meets the efficiency, simplicity, and speed criteria. However, the MF number has a significant impact on the computational time. As a result, the best model for achieving the best system performance can be identified by varying types and MFS. Some MF concepts are literally explored, such as fuzzy logic as a universal approximate or constrained interpolations, which are suitable for MF with finite membership values [61].

The main goal is to separate the 0-1 modelling, which may be accomplished with a triangular MF; however, a more complex situation may necessitate using a specific MF type. A high-fidelity intuition based on sufficient experience can provide a good solution for making the optimal choice. To find memberships for fuzzy variables, MH optimization and evolutionary optimization techniques are used, e.g. NNs and GA. The fuzzy logic approach was used to determine the particle’s performed mutation probability. At any given time, a particle’s mutation probability is not totally certain or uncertain; instead, the membership value provides the mutation probability.

**Fig. 2:**
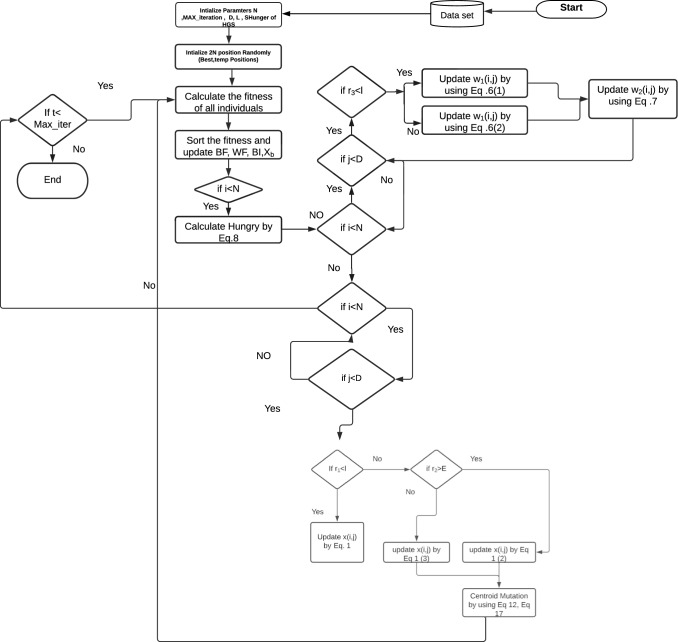
The proposed mHGS algorithm

### Centroid-based fuzzy mutation

This concept is the development of a mutation that aids the population particles in determining when to drift, allowing for premature convergence. There are two crucial parameters to consider with a particle mutation treatment: particles distance from other particles and particles history. As the particle distance from the population centroid is checked, the accuracy of the particles changes. When particles are closer to one another, early convergence is possible. Meanwhile, to avoid this problem, the mutation method is used to separate the particles. This can assist many particles in overcoming the local solution. Estimating population distance from the centroid of other populations is more appropriate than calculating particle distance from other particles. If the distance between populations is small, it means that the populations are close to each other.

The population distance from the centroid is inversely proportional to the mutation probability. In some cases, it is possible that a population lives at the centroid. When the distance is 0, there is an infinite mutation chance, which is undesirable. Therefore, we add one to the distance to ensure that this scenario does not occur. As a result, from Eq. (11) declares the contribution of distance to mutation probability as follows:

11 $$\begin{aligned} P_{d}=\frac{1}{1+\mathrm{{dist}}} \end{aligned}$$

where dist is how much a particle is far from the centroid, P$_{d}$ is approximate contribution, is the distance contribution to the mutation probability. In a similar way to distance, population history may play a role in mutation probability. If the best solution is reached, the frequency of iterations declared by the population will be changed. The best solutions are still explored and trying to find a better solution. But, if the global solution stays static over iterations, this indicates that these solutions are trapped in local solutions and cannot be searched in several parts of the search space. Therefore, mutations in the populations are important to provide some perturbation between them, thereby helping several populations to avoid local optima and find solutions in global optima. If the time for the global best populations is always expanding, raising the mutation probability is also required. This historical information contribution is calculated by ($P_{c}$). The global population has remained unaffected by using the iteration number unchanged for mutation probability. $\alpha =4$ and $\beta =5$, Following these values, the probability of mutation increases as the value of increased.

12 $$\begin{aligned} P_{e}=a+b * \tanh \left( \left( \frac{ \text{ unchanged } }{\alpha }\right) -\beta \right) \end{aligned}$$

where *a* = 0.5 and *b* = 0.5, the tan function returns a number between [1, 1] and [0.5, 0.5] that, when multiplied by *b*, is constrained to [0.5, 0.5]. As a result, the final value is in the range [0, 1]. Equivalent Eq. (12) is used to combine two contributions. Where the parameters $\alpha$ and $\beta$ gave the distance and background contributions equal weight. The values of $\alpha$ and $\beta$ were set to 0.6 and 0.4, respectively. As an example, distance has become increasingly important throughout time. Other particles change, even if the best does not. In this situation, some particles may not be trapped, but the solution will consider the potential for convergence. To avoid this, Eq. (12) assigns a lower weight to history.

13 $$\begin{aligned} P_{l}=\rho * P_{d}+\varphi * P_{c} \end{aligned}$$

Mutation probability Eq. (13) for the $\hbox {i}{\hat{\hbox {t}}}\hbox {h}$ particle in a population is declared by $P_{i}$. If $P_{i}$ is greater than a randomly value generated of particle *i*, the particle is muted, otherwise mutation does not occur. The mutation is done by the two Eqs. (14, 15).

14 $$\begin{aligned}&\varDelta q=0.5 \text{* } \text{ range } *\left( \left( 1-\frac{ \text{ count } }{ \text{ iter } }\right) ^{2}\right) \end{aligned}$$

15 $$\begin{aligned}&\varDelta p=\min \left( \varDelta q, P_{i j}\right) \end{aligned}$$

(1) where $\varDelta p$ indicates the change in the particle’s value in the *j*th dimension, this range denotes the difference between the benchmark function scope’s upper and lower limits, count denotes the current iteration number, iter denotes the total number of iterations to be performed, and $P_{i j}$ denotes the value of the *i*th particle in the *j*th of the entire population dimension. The value of $\varDelta q$ lowers as the points converge, allowing for fewer disruptions. To ensure that the disturbance in an agent’s motion is minimized, Eq. (15), $\varDelta$ is controlled. Despite the fact that there are multiple assumptions in this scenario, the fuzzy mutant forms of the HGS algorithm often have a higher possibility of avoiding the convergence problems.

### Fitness function (fobj)

This section is used to explain the mHGS fitness function (fobj), which is formulated as:

16 $$\begin{aligned}&{\hbox {fobj}}=\alpha +\beta \frac{|R|}{|C|}-G. \end{aligned}$$

17 $$\begin{aligned}&\beta = \alpha \end{aligned}$$

18 $$\begin{aligned} {\hbox {fobj}}>T \end{aligned}$$

where *R* stands for the error rate, *C* stands for the summation of features in the dataset, $\alpha$ and $\beta$ stand for the classification quality importance (as calculated by the classifiers) and subset length, respectively. The range of $\alpha$ is specified as [0, 1]. *G* stands for the classifier’s group column, and *T* stands for the condition that each method is compared to the fitness function. To maximize the solution, fobj must be greater than *T*.

Algorithm 2 describes the steps in the mHGS-SVM algorithm optimization process. In addition, a flowchart depicting the detailed steps of the mHGS procedures is shown in Fig. 2.

**Figure:**
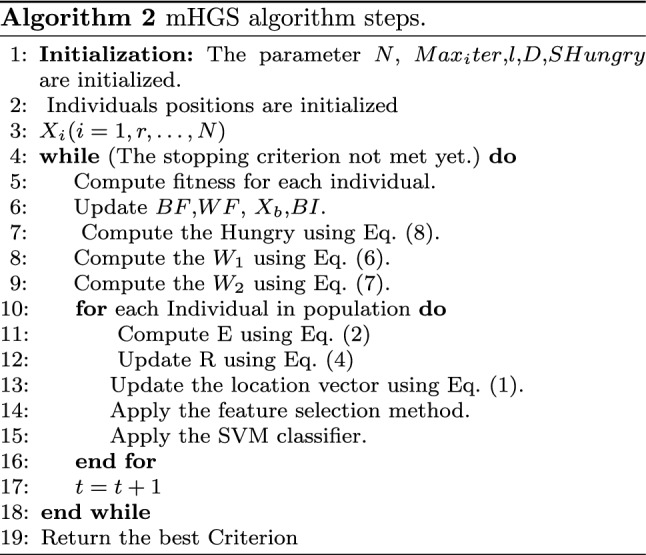

### mHGS development phase

*Initialization stage * The proposed mHGS algorithm starts the optimization process randomly initialize the agents’ population using a uniform random distribution as follows:

19 $$\begin{aligned} X_{i}^\mathrm{{initial}}=LB+{\text {rand}}_{i}\left( {\hbox {UB}}-{\hbox {LB}}\right) \quad i=1,2, \ldots \ldots n \end{aligned}$$

where $X_{i}^{\text{ initial }}$ is the random initialized ${i}{\hat{\hbox {t}}}\hbox {h}$ solution vector, UB, LB are the upper and lower bounds, respectively, *n* is the population size ; ${\text {rand}}_{i} \in [0,1]$ is a random value. The fitness function (fobj ) is calculated using Eq. (16).

*Population reduction* In population-based algorithms, the number of search agents in the population NP plays an essential role in adjusting the algorithm convergence rate. Further explanation: Small population agents can converge quickly but; however, they improve the probability obtaining a local optimum. The population with a large number of agents converges more slowly but provides a better exploration of the search space. The proposed mHGS applies the population reduction approach of linear reduction as follows:

20 $$\begin{aligned} {\hbox {NP}}^{(t+1)}={\text {round}}\left[ \left( \frac{{\hbox {NP}}_{\max }-{\hbox {NP}}_{\min }}{{\hbox {MAX}}_{-} {\hbox {FE}}}\right) * {\hbox {FE}}+{\hbox {NP}}_{\min }\right] \end{aligned}$$

where NP_max_ is the initial population size (NP); NP_min_ is the specified minimum population size ; NP_min_ $=30$ in this study; FE is the current function evaluation; MAX_FE $=45000$ is the maximum number of function evaluations.

*Solution step * Solutions are proposed, and new solutions are proposed using Eqs. (1, 2) as illustrated in Algorithm 2. Applying the fuzzy mutation to improve the search space by exploring several new regions to identify the best candidate solution using Eqs. (14), (15), which helps in improving the diversity of algorithms, and avoid local solutions and comparing between new and previous solution. The best new solution is used for the update using Eq. (6) for hunger and Eq. (7) for calculating the hunger sensation that controls the algorithm performance. Algorithm 2 declares how the best solution is proposed for calculating the objective fitness for several new populations. This process is repeated until the stopping condition is achieved.

*Termination step * The proposed mHGS algorithm is repeated until the stopping criteria are met, resulting in the best candidate solution. The steps of the proposed mHGS algorithm are illustrated in Figure 2, and the pseudo-code is presented in Algorithm 2.

*Classification phase * The best-proposed solution selected in the previous phase is obtained using the mHGS method. In Xbest, the features retrieved from the original dataset are equal to one. The SVM classification approach is implemented. The dataset is split into two sets: 90% train and 10% test. It is worth noting that all experiments were conducted 30 independent times to get the best results.

## Experimental results and simulations

The CEC’20 benchmark functions and FS dataset are applied for evaluating the proposed mHGS including several testing such as statistical results and qualitative metrics. For fair evaluation, the suggested mHGS results were compared with other seven MAs as (IMODE), (GSA), (GWO), (HHO), (WOA), (SMA) and original HGS algorithm. Table 2 shows the parameter settings for all the compared algorithms. To generate results, all of the algorithms were checked using MATLAB programming language. For FS evaluation, all the compared algorithms were hybridized with SVM as a classifier. A total of ten chemical datasets were used. function evaluations (FEs) were utilized each comparative algorithm 30 times with 30 agents.

### Parameter settings and evaluation metrics

Many compared algorithms are used in our experiment to evaluate our enhancement method. Parameter settings have a main role in controlling several conditions in our experiment. All parameters for a fair optimization experiment are defined in Table 2.

**Table 2:** Parameter settings

Algorithms	Parameters setting
Common settings	Search agents: $N=30$
	Dimensions Dim $=10$
	Number of independent runs 30
Centroid mutation	$\alpha =4$, $\beta =5$, $P_{c}=[0,1]$, $a=0.5$, $b=0.5$
IMODE	arch_rate $=2.6$
GSA	alpha $$ = 20, G0 = 100,$$
	Rnorm $= 2$, Rpower $= 1$
GWO	*a* decreases linearly from 2 to 0 (Default)
HHO	$E0 \in [-1, 1]$ (Default)
WOA	$\alpha$ variable decreases linearly from 2 to 0 (Default)
	*a*2 linearly decreases from −1 to −2 (Default)
SMA	$z=0.03$
HGS	VC2 $$ = 0.03$$, sumHungry $$= 0,$$

The following measurements are utilized to validate and evaluate the proposed method depended on the best fitness value fobj obtained at run *i*:

1. The average of the fitness function values produced by running method *M* for times is the mean. The mean fitness function can be calculated using the following equation:
  21 $$\begin{aligned} {\hbox {Mean}} = \frac{ \sum _{i=1}^{M} {{\hbox {fobj}}(i)} }{M} \end{aligned}$$
2. The maximum value of the fitness function obtained by running the algorithm *M* times refers to the best fitness function. The value of the best fitness function can be calculated as follows:
  22 $$\begin{aligned} {\hbox {Best}} = \max _{i=1}^{M} {{\hbox {fobj}}(i)} \end{aligned}$$
3. The worst fitness function is the fitness function with the minimum value produced by performing the algorithm *M* times. The value of the worst fitness function can be computed by
  23 $$\begin{aligned} {\hbox {Worst}} = \min _{i=1}^{M} {{\hbox {fobj}}(i)} \end{aligned}$$
4. Standard deviation is used to measure the fluctuation of the fitness function value obtained from *M* times of the running algorithm (STD). STD is an indication used to measure the stability and robustness of the algorithm. Higher standard deviation values suggest that the algorithm wanders, but a smaller number shows that the method converges for the same value in the majority of iterations. Using the formula below, the standard deviation can be calculated:
  24 $$\begin{aligned} {\hbox {STD}} = \sqrt{\frac{1}{M-1}\Sigma _{{i=1}}^{M} ({\hbox {fobj}}(i)-{\hbox {mean}}) ^2} \end{aligned}$$

### Experimental series 1: statistical results for CEC’20

To assess the proposed mHGS approach, the IEEE Congress on Evolutionary Computation 2020 (CEC’20) [62] was selected for evaluating its performance while solving different types of suite objective functions. Statistic methods are applied to algorithms that use CEC’20 functions to indicate which algorithm is the best.

#### CEC’20 benchmark functions description

The test data for testing the performance of the suggested algorithms were taken from the IEEE Congress on Evolutionary Computation (CEC) [62]. Ten test functions were included in the CEC’20 benchmark functions, containing unimodal, multimodal, hybrid, and composition functions.

#### Parameter space

**Fig. 3:**
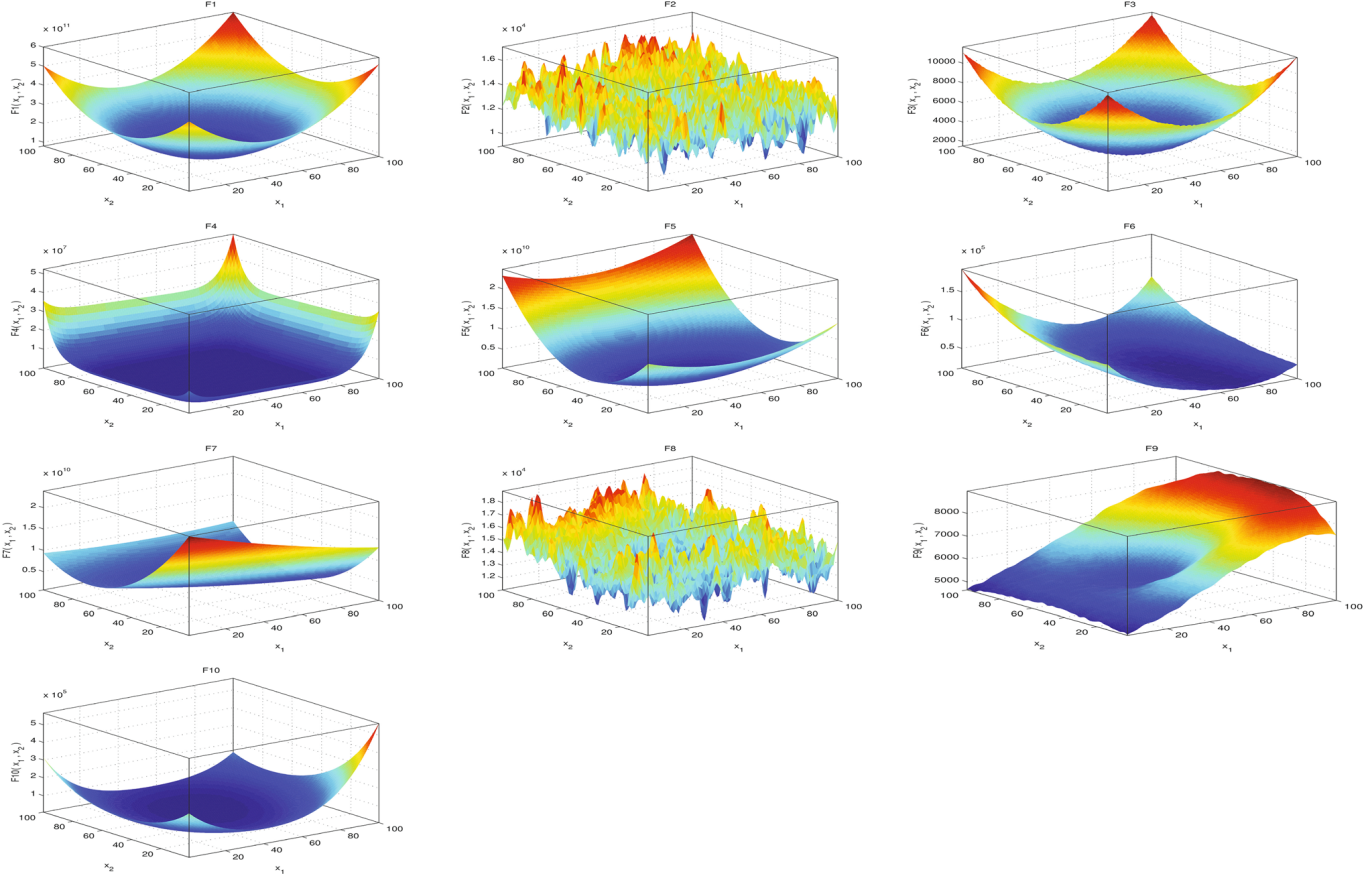
The 2D visualization of the CEC’20 benchmark functions

Figure 3 declares a 2D visualization of the CEC’20 functions to help understanding the differences and each problem nature.

#### Statistical results analysis

Table 3 reports the statistical results such as mean and STD for the proposed algorithm and other compared algorithms for each CEC’20 benchmark function with 10-dimension; the best results (minimum values). The suggested approach outperformed previous algorithms in solving most of the CEC’20 benchmark functions in terms of mean and standard deviation. Furthermore, the proposed mHGS was ranked top in the Friedman mean rank-sum test.

**Table 3:** The mean and standard deviation terms for 30 runs with Dim $=10$

Functions	IMODE		GWO		GSA		WOA		HHO		SMA		HGS		mHGS	
	Mean	STD	Mean	STD	Mean	STD	Mean	STD	Mean	STD	Mean	STD	Mean	STD	Mean	STD
F1	1.01E+02	6.70E−15	1.00E+05	1.89E+05	7.67E+02	7.39E+02	2.13E+06	1.63E+06	6.67E+05	2.86E+05	6.84E+03	4.25E+03	1.28E+06	6.07E+05	1.00E+02	2.71E−05
F2	1.90E+03	4.25E−01	1.90E+03	7.07E−01	1.90E+03	3.30E−01	1.91E+03	2.97E+00	1.91E+03	2.25E+00	1.90E+03	7.57E−01	1.90E+03	5.88E−01	1.30E+03	1.78E−02
F3	1.73E+03	3.30E+01	6.05E+04	1.77E+05	5.34E+05	1.75E+05	1.68E+05	2.56E+05	2.57E+04	2.74E+04	6.54E+03	5.90E+03	1.95E+03	8.23E+01	7.18E+00	3.42E+00
F4	1.60E+03	9.87E−02	1.61E+03	9.08E+00	1.64E+03	1.98E+01	1.61E+03	7.48E+00	1.62E+03	9.49E+00	1.60E+03	2.31E−01	1.60E+03	1.51E−01	1.90E+02	5.68E−03
F5	2.10E+03	6.94E+00	8.82E+03	5.51E+03	4.10E+05	3.57E+05	9.26E+04	1.07E+05	1.59E+04	1.23E+04	5.04E+03	3.73E+03	2.17E+03	6.88E+01	1.82E+03	5.64E+01
F6	2.29E+03	2.46E+01	2.35E+03	1.27E+02	2.30E+03	2.54E−01	2.45E+03	4.27E+02	2.31E+03	4.06E+00	2.31E+03	3.57E+01	2.29E+03	3.63E+01	1.60E+03	2.15E−01
F7	2.55E+03	1.02E+02	2.75E+03	9.87E+00	2.67E+03	1.44E+02	2.77E+03	9.71E+01	2.81E+03	1.13E+02	2.75E+03	6.66E+00	2.75E+03	8.09E+00	2.19E+03	8.79E+01
F8	2.92E+03	2.28E+01	2.94E+03	2.61E+01	2.93E+03	2.15E+01	2.94E+03	1.94E+01	2.93E+03	2.36E+01	2.95E+03	3.12E+01	2.91E+03	2.01E+01	2.30E+03	2.30E+00
F9	2.54E+03	1.09E+02	2.74E+03	1.15E+01	2.65E+03	1.27E+02	2.76E+03	5.75E+01	2.81E+03	1.24E+02	2.76E+03	6.12E+00	2.75E+03	1.06E+01	2.08E+03	1.31E+00
F10	2.93E+03	2.29E+01	2.94E+03	1.49E+01	2.94E+03	1.38E+01	2.95E+03	1.18E+01	2.92E+03	2.44E+01	2.93E+03	2.61E+01	2.94E+03	3.77E+01	2.91E+03	2.31E+01
Friedman mean rank	4.5		2.3		6.8		4.7		6.8		6.2		3.3		1.4	
Rank	4		2		7		5		8		6		3		1	

#### Convergence behaviour analysis

**Fig. 4:**
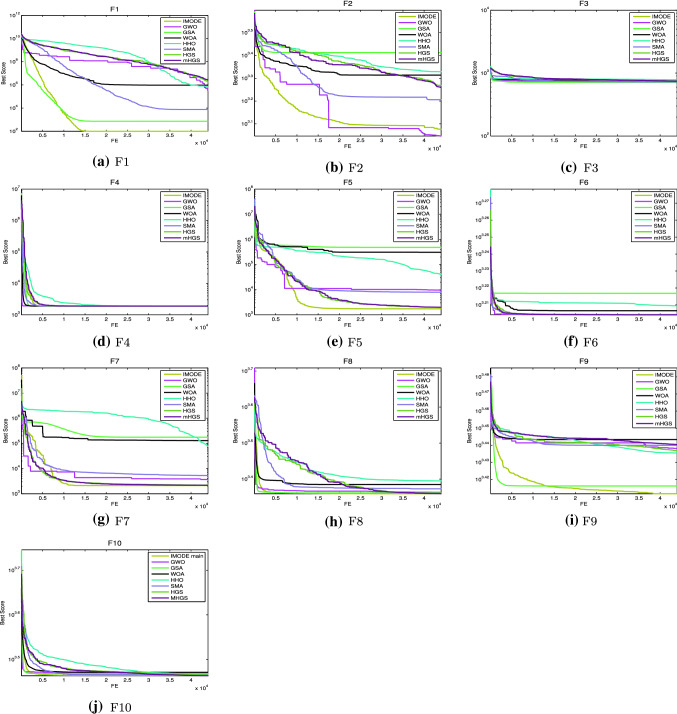
Convergence curves for the proposed mHGS and other comparison algorithms on CEC’20 functions Dim $=10$

The mHGS algorithm and other algorithm performance can be explained with convergence curves and counterparts with the CEC’20 functions shown in Fig. 4. All the results of the functions for the proposed algorithm reached a stable point, indicating that, it converges. Fast convergence indicates the optimal solution. Thus, the suggested mHGS method is a promising optimization to solve several problems that need fast computing, such as online optimization problems.

#### Boxplot behaviour analysis

**Fig. 5:**
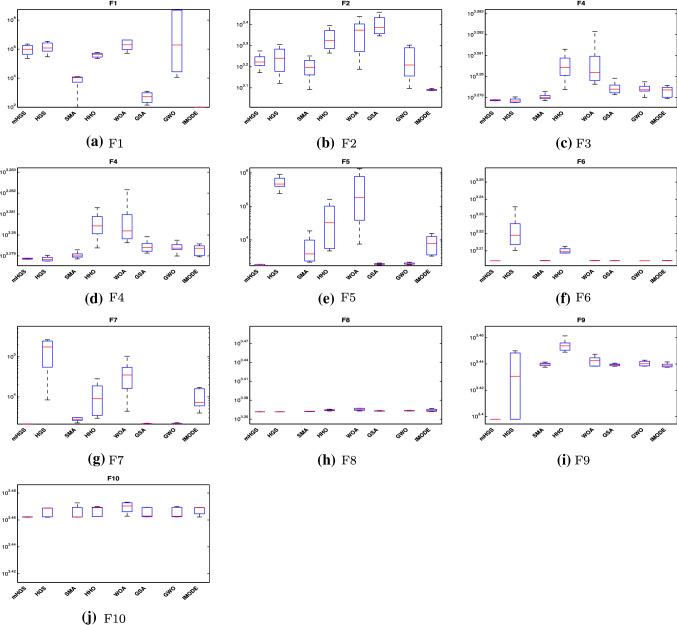
The boxplot curves of the proposed mHGS and other comparsion algorithms on CEC’20 functions with Dim $=10$

Boxplots are used to show data distribution characteristics. The distribution results are related to several local minima of class functions, as shown in Fig. 5. Boxplots are effective for presenting data distributions in quartiles. The algorithm’s minimum and maximum data points, which are the whisker’s edges, are the algorithm’s lowest and highest data points. High level of data agreement is declared by a narrow boxplot Fig. 5. Boxplot shows the results for ten functions Dim $=10$. The mHGS algorithm produces the best results when compared to other algorithms.

#### Qualitative metrics analysis

**Fig. 6:**
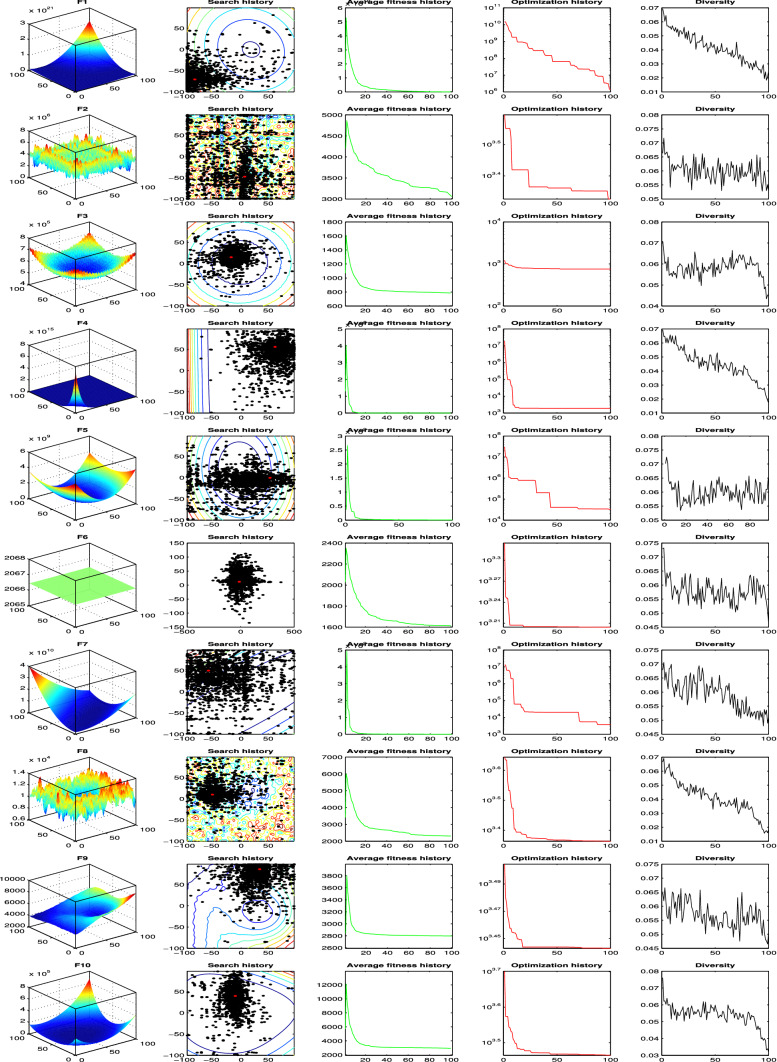
The qualitative metrics on CEC’20 benchmark functions: 3D views of the functions, search history, average fitness history, and optimization history

Particle behaviour monitoring, or search agents, for instance, can propose more knowledge about algorithm convergence and the optimization search process. The mHGS algorithm qualitative analysis is indicated in Fig. 6. The agent’s behaviours are shown in Fig. 6, which illustrates the functions in two dimensions (3D), search history, average fitness history, and convergence curves.

*These points discuss the qualitative analysis*

- *In terms of domain’s topology—functions in 3D views * The function in 3D space is indicated in the first column of Fig. 6. The functions have distinct topologies, which aids in deciding which type or shape of function the algorithm performs best.
- *For the search history * The search history of agents is shown in the second column of Fig. 6 from the first to the last iteration. The search space is represented by counter lines, with the gradient from blue to red lines indicating a higher fitness value. The suggested mHGS can locate the locations with the lowest fitness values for particular functions, according to search history.
- *In terms of average fitness history * The average fitness history is shown in the third column of Fig. 6, i.e. the fitness value averages as a function of the iteration number. This average reveals the agents’ overall behaviour as well as their contribution in the optimization process. The population improves as the history curves diminish. This continuous improvement demonstrates a collaborative searching behaviour and backs up the efficacy of particle law updates.

### Experimental series 2: applying mHGS for FS

#### Data description

We have used several datasets that was collected from Machine Learning Repository and kaggle websites but only MAO data set taken from GREYC’s Chemistry dataset.^1^,^2^,^3^*Monoamine Oxidase (MAO)* The dataset is provided by an enzyme that is widely distributed in the major tissues. It has the ability to catalyse the inactivation and oxidation of monoamine neurotransmitters. The GREYC Chemistry dataset provided the data for this dataset. This data set is taken from https://brunl01.users.greyc.fr/CHEMISTRY/#MAO. Thus, MOA is transferred to SMILES (Simplified molecu//-lar-input lineen try system) styles using open babel software [63]. Then, the molecular descriptors (MD) are determined using E-dragon [64]. It has 1665 features (MD) with 68 compounds divided into two classes.

*QSAR Biodegradation * This dataset has 41 features (molecular descriptors) that are used to classify 1055 chemical compounds. These data are used to determine between two chemical classes, with 356 readily biodegradable samples and 699 not readily biodegradable patterns. Furthermore, this information can be used in the building of QSARs to indicate the relationship between molecular biodegradation and chemical design. It is available on the UCI Web page (https://archive.ics.uci.edu/ml/datasets/QSAR+biodegradation).

*Drug Review * This dataset includes patient reviews for specific medicines as well as diseases that are related to them. It has ten patients who have given it a rating that reflects overall patient satisfaction. Crawling online pharmaceutical review sites yielded the information. The goal was to learn something new. Splitting this data into a train (75%) and a test (25%) yields the greatest results (25%). It is available on the UCI Web page (https://archive.ics.uci.edu/ml/datasets/Drug+Review+Dataset+%28Drugs.com%29).

*Drug consumption * There are 1885 responders in the database. All input attributes are categorical at first and are then quantified. Participants were also asked about their use of 18 legal and illegal drugs, including alcohol, amphetamines, benzodiazepine, cannabis, chocolate, cocaine, caffeine, crack, ecstasy, heroin, ketamine, legal highs, LSD, methadone, mushrooms, nicotine, and volatile substance abuse, as well as one fictitious drug to identify over-claimers. It is available on the UCI Web page (https://archive.ics.uci.edu/ml/datasets/Drug+consumption+%28quantified%29).

*QSAR androgen receptor * Using various machine learning methods, this dataset was used to create classification QSAR models for the discrimination of binder/positive (199) and non-binder/negative (1488) molecules. The following reference provides more information: Machine Learning Consensus to Predict the Binding to the Androgen Receptor within the CoMPARA Project, 59, 1839-1848, Journal of Chemical Information and Modeling. The Milano Chemometrics and QSAR Research Group (Universit degli Studi Milano-Bicocca, Milano, Italy) calculated attributes (molecular fingerprints) on a set of chemicals provided by the National Center of Computational Toxicology at the US Environmental Protection Agency as part of the CoMPARA collaborative modelling project, which aimed to develop QSAR models to identify binders to the Androgen Receptor. It is available on the UCI Web page (https://archive.ics.uci.edu/ml/datasets/QSAR+androgen+receptor).

*Immunotherapy * This dataset contains 90 instances of wart treatment results and has 8 number of attributes. It is available on the UCI Web page (https://archive.ics.uci.edu/ml/datasets/Immunotherapy+Dataset).

*Anticancer Peptides * Membranoid anticancer peptides (ACPs) are drawing increasing as potential cancer therapies due to their ability to prevent cellular resistance and overcome common hurdles such as chemotherapy side effects and cytotoxicity. The anticancer action of peptides (annotated for their one-letter amino acid code) on breast and lung cancer cell lines is described in this dataset. It is of a high standard (active, moderately active, experimental inactive, virtual inactive). It is available on the UCI Web page (https://archive.ics.uci.edu/ml/datasets/Anticancer+peptides).

*Gene Expression Cancer RNA-Seq * The samples are sorted by row. The RNA-Seq gene expression levels measured by the illumina HiSeq platform are the attributes of each sample. Number of attribute is 20531 for 801 sample, it is available on the UCI Web page (https://archive.ics.uci.edu/ml/datasets/gene+expression+cancer+RNA-Seq).

*Primary Tumour * This is one of three domains provided by the Oncology Institutenthat has repeatedly appeared in the machine learning literature. For 339 instances, it contains 17 attributes. It is available on the UCI Web page (https://archive.ics.uci.edu/ml/datasets/primary+tumor).

*Alzheimer Features * This dataset is used to describe Alzheimer features. It consists of 347 instances with 10 features. This dataset is collected from kaggle website’s Web page (https://www.kaggle.com/datasets/brsdincer/alzheimer-features).

*Eye Disorder * This dataset discusses eye disorder. It is used to describe 101 instances for 16383 features. It is available on the kaggle website’s Web page (https://www.kaggle.com/datasets/prateek0x/eye-disorder-dataset).

#### Data pre-possessing

**Fig. 7:**
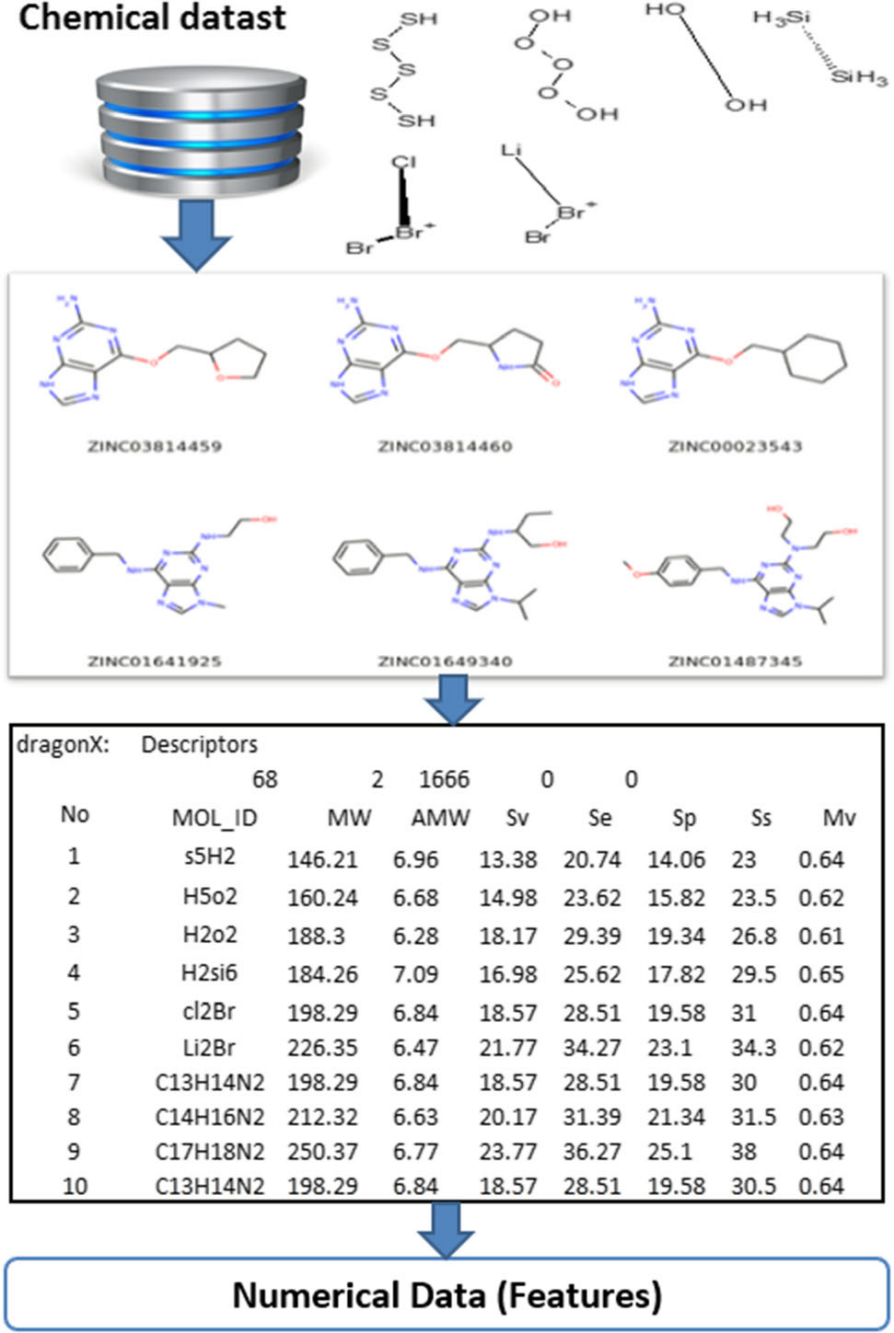
Mapping from molecules to features

Some chemical data may require some preprocessing procedures (Fig. 7) which illustrates the main stages of the preparation process: (1) The information about proteins is transformed into a chemical representation; (2) descriptors are calculated; and (3) the chemical structure is converted into a mathematical form. The following are the phases.

**Fig. 8:**
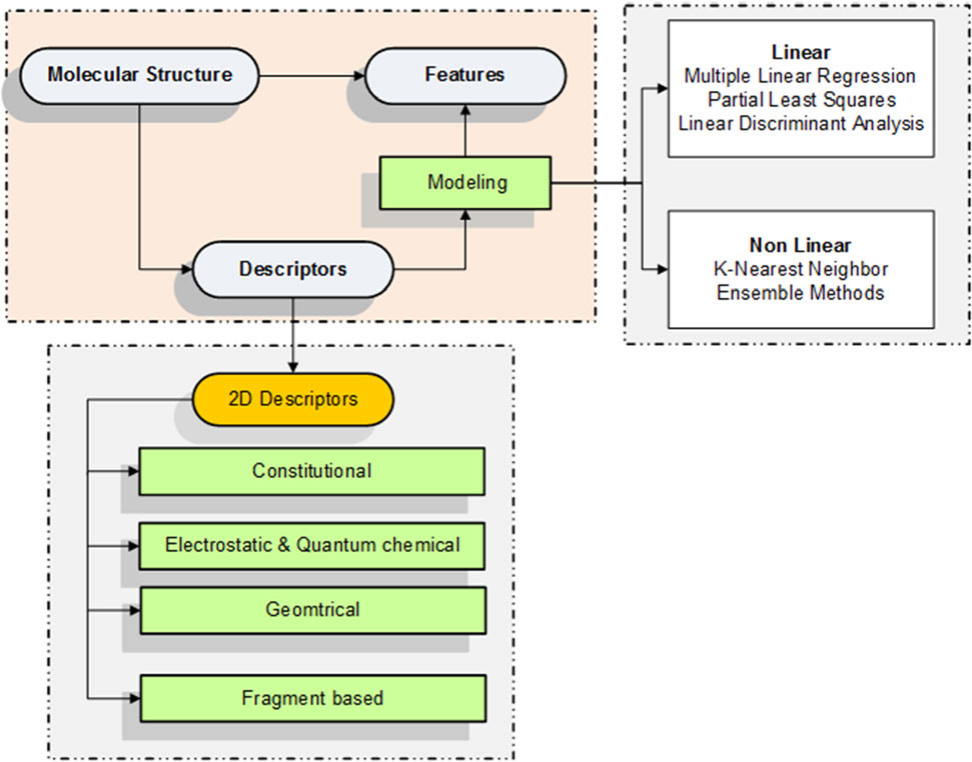
Flowchart of QSAR model

1. Protein information is transformed into an isomeric simplified molecular-input line entry system (SMILES) using the open Babel software [63]. The information about proteins is stored in a chemical format, called MOA, which must be transformed into isomeric SMILES using Babel software. Features are attributes with values used to create instances.
2. E-Dragon is used to calculate the descriptors, and several chemical features are performed to implement several 2*D* and 3*D* data in the QSAR model and calculate descriptors using E-Dragon software. The descriptors are divided into three categories: rotary links, structural, and physicochemical links (weight and volume of molecule, distance between atoms, atom type, molecular account walking, electronegativity, atom distribution, aromatic, and thawed characteristics).
3. The QSAR model expresses the mathematical relationship between chemical design and biological activity. Additionally, the features can be used to identify the instances. As shown in Fig. 8, QSAR is used to declare the major properties of chemical compounds. Moreover, structure-activity correlation analysis uses different ML algorithms to predict the similarity of chemicals in the presence of a disease. Compounds of complex molecules have many characteristics, such as topological factors [52].

#### Statistical results analysis

Table 3 presents the statistical criteria of the best value that was provided from the suggested algorithm mHGS compared with other algorithms for each dataset. Results in terms of mean, STD, best, worst, and CPU time values revealed the preference of the proposed algorithm in solving the FS problem compared to other algorithms. The best is the maximum value, the worst is the minimum, and STD is the smallest value which are depicted in Tables 4 and 5.

**Table 4:** Mean, STD, Best, Worst and Computational time values obtained by the selected algorithms using svm and a stop criterion based on FE

Algorithm	Mean	STD	Best	Worst	CPU time
*Dataset no. 1: monoamine oxidase (MAO)*					
GWO	9.00E+01	1.08E−01	90.231	89.998	0.741
WOA	9.10E+01	9.13E−01	90.713	88.973	0.936
GSA	9.20E+01	3.27E−01	91.190	90.100	0.729
HHO	9.69E+01	3.09E−02	95.093	94.180	0.480
HGS	9.19E+01	4.86E−02	97.199	94.701	0.761
mHGS	9.80E+01	4.37E−03	98.034	95.405	0.860
SMA	8.42E+01	4.40E−00	90.054	88.144	0.811
IMODE	8.57E+01	4.39−E00	88.954	87.785	0.991
*Dataset no. 2: QSAR biodegradation*					
GWO	9.00E+01	1.08E−01	85.130	84.190	0.620
WOA	9.01E+00	9.01E−01	86.010	85.120	0.936
GSA	9.20E+01	3.07E−00	88.091	87.091	0.707
HHO	9.66E+01	3.07E−02	89.090	88.182	0.401
HGS	9.19E+01	4.86E−02	90.190	89.700	0.761
mHGS	9.60E+01	2.07E−03	92.001	91.115	0.700
SMA	8.41E+01	4.20E−00	88.150	87.130	0.800
IMODE	8.10E+01	3.30−E00	86.920	85.180	0.970
*Dataset no. 3: drug review*					
Drug review GWO	8.75E+00	1.07E−01	87.010	86.071	0.600
WOA	8.70E+00	1.00E−00	85.100	84.120	0.910
GSA	8.72E+01	1.17E−00	86.190	85.190	0.177
HHO	8.90E+00	1.10E−02	88.120	87.081	0.410
HGS	9.00E+01	1.86E−02	90.090	89.712	0.360
mHGS	9.10E+01	1.90E−02	91.909	90.010	0.720
SMA	8.90E+01	1.21E−00	89.052	88.100	0.860
IMODE	8.40E+01	1.00−E00	85.021	84.081	0.961
*Dataset no. 4: drug consumption*					
GWO	8.85E+00	1.07E−01	89.112	88.170	0.600
WOA	8.75E+00	1.00E−00	88.112	87.101	0.911
GSA	8.70E+01	1.17E−00	87.010	86.010	0.078
HHO	9.50E+00	1.70E−02	90.021	89.180	0.400
HGS	9.60E+01	1.89E−02	91.191	90.110	0.300
mHGS	9.70E+01	1.99E−02	93.100	91.011	0.700
SMA	8.80E+01	1.21E−00	88.150	87.120	0.800
IMODE	8.60E+01	1.00−E00	87.120	86.080	0.900
*Dataset no. 5: QSAR androgen receptor*					
GWO	9.05E+01	1.29E−01	90.110	89.100	0.620
WOA	9.00E+00	1.10E−01	89.100	88.109	0.901
GSA	8.90E+01	1.77E−01	88.019	87.210	0.028
HHO	9.10E+00	1.80E−02	92.220	90.100	0.410
HGS	9.20E+01	1.99E−02	93.190	92.100	0.300
mHGS	9.40E+01	2.00E−02	94.160	92.010	0.720
SMA	8.80E+01	1.21E−00	88.150	87.120	0.820
IMODE	8.60E+01	1.20−E00	87.120	86.080	0.901

**Table 5:** Mean, STD, Best, Worst and CPU time values obtained by the selected algorithms using the svm and a stop criterion based on FE

Dataset	Algorithm	Mean	STD	Best	Worst
*Dataset no. 6: immunotherapy*					
GWO	9.45E+01	1.90E−02	92.121	90.121	0.600
WOA	9.40E+00	1.91E−01	90.121	89.120	0.800
GSA	8.95E+01	1.79E−01	89.110	86.011	0.023
HHO	9.350E+00	1.89E−02	93.100	91.121	0.400
HGS	9.40E+01	2.19E−02	94.091	92.101	0.201
mHGS	9.50E+01	2.20E−02	95.061	94.112	0.621
SMA	8.90E+01	1.91E−01	88.150	87.120	0.721
IMODE	8.70E+01	1.99−E00	87.021	86.183	0.801
*Dataset no. 7: anticancer peptides breast cancer*					
GWO	9.35E+01	1.80E−02	90.020	89.120	0.300
WOA	9.33E+00	1.81E−01	89.020	88.100	0.806
GSA	8.99E+01	1.72E−01	87.010	86.010	0.020
HHO	9.40E+00	1.99E−02	91.120	90.101	0.200
HGS	9.50E+01	2.29E−02	92.190	91.101	0.100
mHGS	9.55E+01	2.40E−02	93.160	92.110	0.600
SMA	8.99E+01	1.98E−01	88.150	87.120	0.700
IMODE	8.80E+01	1.90−E00	86.001	85.180	0.600
*Dataset No. 8: Gene expression cancer RNA-Seq*					
GWO	9.75E+01	1.89E−02	94.122	93.021	0.700
WOA	9.70E+00	1.86E−01	93.130	92.122	0.900
GSA	8.99E+01	1.79E−01	89.211	88.112	0.600
HHO	9.80E+00	2.09E−02	96.100	95.100	0.300
HGS	9.90E+01	2.50E−02	97.010	96.700	0.500
mHGS	9.95E+01	2.70E−02	98.060	97.041	0.900
SMA	8.90E+01	1.80E−01	89.121	88.100	0.800
IMODE	8.85E+01	1.99−E00	87.100	86.200	0.700
*Dataset No. 9: Anticancer peptides lung cancer*					
GWO	9.75E+01	1.40E−02	92.120	90.020	0.200
WOA	9.65E+00	1.60E−01	91.030	90.120	0.300
GSA	8.50E+01	1.70E−01	90.210	89.110	0.400
HHO	9.70E+00	1.45E−02	93.120	92.140	0.200
HGS	9.80E+01	1.50E−02	94.112	93.710	0.500
mHGS	9.85E+01	1.79E−02	95.261	94.140	0.300
SMA	8.95E+01	1.90E−01	89.920	88.900	0.300
IMODE	8.75E+01	1.79−E00	88.120	87.900	0.400
*Dataset No. 10: Alzheimer features*					
GWO	9.75E+01	1.40E−02	89.021	88.021	0.100
WOA	9.65E+00	1.60E−01	88.130	87.100	0.200
GSA	8.50E+01	1.70E−01	89.110	88.100	0.300
HHO	9.70E+00	1.45E−02	91.021	90.100	0.100
HGS	9.80E+01	1.50E−02	92.010	91.511	0.200
mHGS	9.85E+01	1.79E−02	93.200	92.041	0.400
SMA	8.95E+01	1.90E−01	88.721	87.920	0.200
IMODE	8.75E+01	1.79−E00	87.100	86.950	0.300

#### Convergence behaviour analysis

**Fig. 9:**
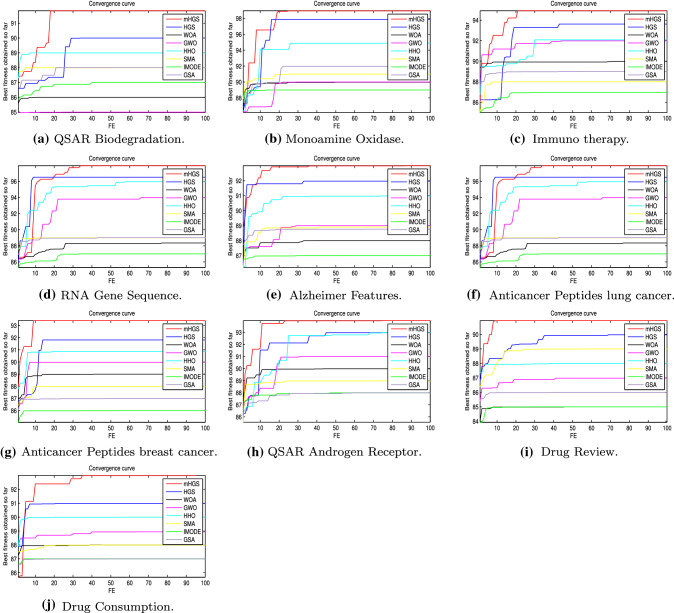
The convergence curves obtained from the proposed mHGS and the competitor algorithms over ten datasets

The convergence curves of the proposed mHGS have been compared to other algorithms. The comparison is presented in Fig. 9 for ten datasets. mHGS algorithm has a stable point for all datasets, suggesting that the proposed algorithm converges. Furthermore, mHGS has achieved the greatest average of the best solutions, the fastest for most datasets. The suggested mHGS algorithm is a promising optimization choice for solving FS problems and achieving high accuracy when compared to existing algorithms, as shown in Fig. 9.

#### Boxplot behaviour analysis

**Fig. 10:**
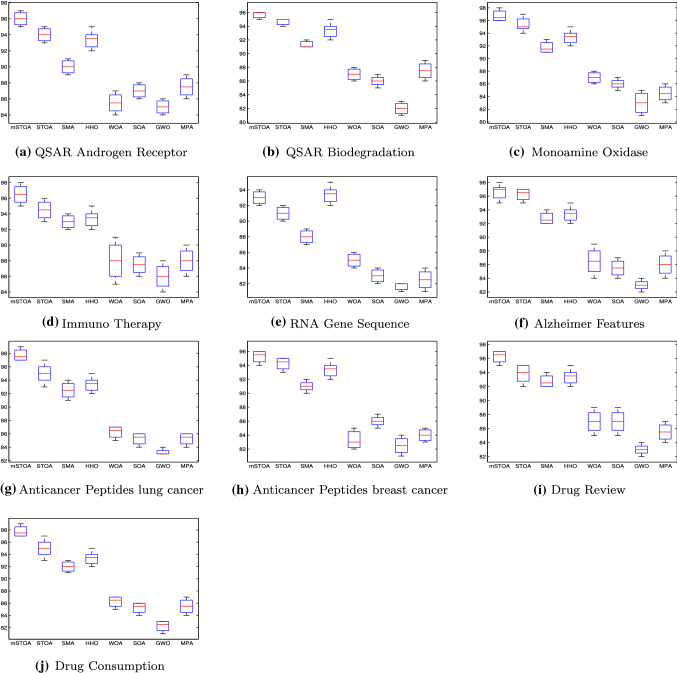
The boxplots comparison obtained from the counterparts algorithms using SVM on the different stop criteria applied over ten datasets

The boxplot is used to evaluate the performance of several dataset as a non-parametric method. However, in descriptive statistics, a boxplot is a way for graphically depicting groups of numerical data through their quartiles. Boxplots may also have lines extending vertically from the boxes, indicating variability outside the upper and lower quartets; thus, the terms “box-and-whisker plot” and “box-and-whisker diagram”. The maximum or minimum is the largest or the lowest data points achieved by the algorithm. Individual points can be plotted as outlines. The distance between the various parts of the box reflects the degree of spread and skewness in the data, as well as the contours of the data. In the experiments, the boxplots for mHGS-SVM over the ten datasets are presented in Fig. 10. The boxplots of the proposed mHGS algorithm are very narrow compared to other algorithm distributions for most datasets.

### Discussion

Firstly, the proposed mHGS and other compared algorithms are assessed on the CEC’20 benchmark. After that, ten medical dataset are used to evaluate the proposed mHGS-SVM performance. For the CEC’20 benchmark, quantitative and qualitative metrics are used to assess mHGS performance. The proposed mHGS has achieved the highest value, but the IMODE algorithm achieved the lowest results for mean and STD statistical results, as shown in Table 3 and the best for convergence. The minimum convergence curve and boxplot as drawn in Figs. 4 and 5. Figure 3 shows the parameter space is used for 3D visualization of the CEC’20 functions to understand the differences and nature of each problem. The qualitative metrics are used to draw stronger conclusions regarding the algorithm performance for a real problem to confirm the high performance of the proposed mHGS algorithm as shown in Fig. 6.

For FS, the proposed mHGS-SVM maximizes accuracy and reduces the number of features. The mHGS-SVM achieved the best value for mean, STD, best, worst, and computational time as shown in Tables  4 and 5, over all the datasets. The evidence for this fact is supported by the convergence curves when it is possible to see that the mHGS-SVM over ten medical dataset, as illustrated in Fig. 9. The convergence curve is selected because it represents the relationship between the number of features and the fitness function. It indicates the best-performed algorithm from the comparison between different approaches. Boxplot analysis indicates that mHGS-SVM achieved the highest performance compared with other algorithms, as shown in Fig. 10.

According to the above analysis, the proposed mHGS-SVM approach has reached better results than other counterparts. The HGS is the second-ranked, whereas the IMODE is the last rank. For clear comparison and under the same parameter setting, the search agents number was set to 30 for all experiments with a different number of dimensions, according to different dataset dimensions.

### Comparison with existing studies

This subsection will go over several algorithms for automating fuzzy modelling. Many algorithms can be used to automate fuzzy modelling. The comparison of MHs fuzzy logic algorithms is described in Table 6. When choosing meta-heuristic algorithms for fuzzy modelling, there are many elements to consider. Many elements play an important role to the algorithm’s ability, including the representation of fuzzy parameters, the interpretability of the fuzzy model produced by the algorithm. In addition to the algorithm’s parameters, such as population number and the specific parameters according to the algorithm itself, the process involved in the algorithm, and the algorithm’s processing speed. A comparison analysis of MHs to determine the strengths and drawbacks of these algorithms is shown in Table 7.

**Table 6:** MHs depended on fuzzy logic

References	Year	Algorithm	The proposed model	Contribution
[48]	2016	Fuzzy logic controller with GA optimization	This paper described rear-end collation control, which regulates a car’s movement using a fuzzy logic framework. The fuzzy rules were then improved with GA to keep the model’s accuracy while reducing its complexity. The method has been validated using simulation	Rear-end collisions number is minimized and system efficiency is increased
[65]	2016	DE based Fuzzy Logic Controller (FLC)	In the fuzzy system, there were two levels. The first level will search for the fuzzy set’s decreased and increased values to generate the colour delay. Meanwhile, the DE was applied to reshape and optimize the second-level membership function. The suggested technique was put to the test in nine traffic scenarios	In traffic light signal control, boosting the membership function
[66]	2018	GSA-based fuzzy control	To work with a nonlinear control beam system, a fuzzy system was applied. The controller parameters were fine-tuned using GSA	In beam control, improving the performance of fuzzy systems
[67]	2020	Gravitational Search Algorithm (GSA) with fuzzy system	A fuzzy approach was used in the categorization process, and GSA was enhanced to become Comprehensive CLGAS	The CLGAS used to obtain the optimal solution to detect breast tumour
[68]	2018	PSO with the fuzzy concept	To create the best BLDCM system, the fuzzy principle was utilized to model DC motor activity, and the PSO was applied to optimize the DC motor model	Optimizing the BLDCM system

**Table 7:** Comparing various MHAs

Algorithm	Advantages	Problems
(DE) Algorithm	The comparison with other EA algorithms so, DE is faster at finding the solution and needs less calculation time [69]	As a result of, when global and local search is imbalanced, the DE can become trapped in local optima, decreasing precision and speed of convergence [70]
Genetic Algorithm (GA)	Programmability is both efficient and simple. This is the case because chromosomes can be represented as bit strings. This streamlines the solution representation process by simplifying the representation procedure [71]. The GA is the most effective method when the search space is large and complex	Working with dynamic datasets is difficult [72]. Because mutation and crossover are done at random, it has a significant amount of time finding the global optimum, is trapped in the local optimum, and has a high rate of early convergence [73]
Gravitational Search Algorithm	The GSA is a straightforward algorithm with a simple concept [74]. The GSA performs well in global searches due to its fast exploration [75] GSA is a memory-free technique with an adjustable learning rate [74]	GSA search method is extremely slow, which has an impact on exploitation ability and convergence rate [76]. Slow convergence and a tendency to become trapped in local minima [77].The GSA ability to do local searches is limited, and its convergence speed is slow, due to the lack of a local search mechanism
ACO Algorithm	Because it has guaranteed convergence, the ACO can find the best solution [73]. Because the ACO is based on a graph, the search space solution is less redundant [78]	The ACO is difficult to implement and analyse theoretically [79]

## Conclusion and future directions

In this work, an approach to improve the original hunger games search algorithm (HGS) is offered by employing the fuzzy mutation method and linear reduction exploration to avoid local optimal and improve the balance between global and local search. The modified mHGS algorithm was tested on the standard benchmark CEC’20. mHGS was applied for a classification strategy and FS method in several biomedical datasets. The SVM method was used for classifying the data, and it had a high average accuracy rate of 98.060%. Furthermore, using the mHGS for FS significantly improved the SVM classification performance. The experimental results showed that the proposed method provides superior classification results than others. In the future studies, a multi-objective optimization algorithm will be implemented for HGS to solve the FS problem in high-dimensional biomedical datasets.

As future directions, the proposed mHGS algorithm can be utilized in the following future perspectives such as (1) solving other real-world and large-scale optimization problems, (2) solving different engineering and real-world problems with unknown search spaces, (3) tackle with different problems such as feature selection, parameter identification, and task scheduling, and (4) solving multi-objective problems can be investigated in the future studies.

## Data Availability

Data sharing is not applicable to this article as no datasets were generated or analysed during the current study.

## Footnotes

1 https://brunl01.users.greyc.fr/CHEMISTRY/.

2 https://archive.ics.uci.edu/ml/index.php.

3 https://www.kaggle.com/.
